# Practice and associated factors of traditional uvulectomy among caregivers having children less than 5 years old in South Gondar Zone, Amhara Region, Ethiopia, 2020

**DOI:** 10.1371/journal.pone.0279362

**Published:** 2022-12-22

**Authors:** Berhanu Wale Yirdaw, Mengistu Berhanu Gobeza, Netsanet Tsegaye Gebreegziabher

**Affiliations:** 1 Department of Pediatrics and Child Health Nursing, Teda Health Science College, Gondar, Ethiopia; 2 Department of Pediatrics and Child Health Nursing, College of Medicine and Health Sciences, University of Gondar, Gondar, Ethiopia; 3 Department of Emergency and Critical Care Nursing, College of Medicine and Health Sciences, University of Gondar, Gondar, Ethiopia; Jiangsu University, CHINA

## Abstract

**Introduction:**

Traditional uvulectomy is widely practiced in Africa especially in sub-Saharan countries including Ethiopia. Studies conducted in different times and areas of the world have shown that the level of practice of uvulectomy and its associated factors were varied from country to country. Therefore, this study was carried out to assess the practice and associated factors of traditional uvulectomy among caregivers having children less than 5 years old in the South Gondar Zone.

**Objective:**

This study aimed to assess practice and associated factors of traditional uvulectomy among caregivers having children less than 5 years old in South Gondar Zone, Amhara Region, Ethiopia, 2020.

**Method:**

A community–based cross-sectional study was conducted on 634 participants who were selected using a multistage with a simple random sampling method. Data were collected using a structured interviewer-administered Amharic version questionnaire; it was entered into Epi Data and analyzed using SPSS. Descriptive statistics were calculated and logistic regressions were fitted to declare statistical significance at p-value < 0.05 and 95% CI.

**Result:**

The prevalence of traditional uvulectomy in this study was 52.5% (95% CI, 48.6–56.3%). Moreover, lack of information [AOR = 2.975 (1.677–5.277)], perceived as uvula causes illness [AOR = 4.888 (2.954–8.086)], future intention or will perform [AOR = 4.188 (2.584–6.788)], perceived as traditional uvulectomy should not be eradicated [AOR = 1.893 (1.172–3.057)]), saw the previous good result [AOR = 9.396 (5.512–16.016)], health personnel hospitality problem [AOR = 5.922 (2.392–14.664)] and did not get cured by pharmacologic treatment [AOR = 3.918 (2.073, 7.405)] were significantly associated with traditional uvulectomy.

**Conclusion and recommendation:**

The prevalence of traditional uvulectomy was high. Lack of information, perceived as uvula causes illness, future intention to uvulectomy, perceived as traditional uvulectomy should not be eradicated, saw the previous good result, health personnel hospitality problem and did not get cured by pharmacologic treatment were the factors significantly associated with traditional uvula cutting. Therefore, special attention will be given to creating further awareness to the community at large and setting controlling mechanisms for the health care delivery system.

## Background

Uvulectomy is the entire or partial removal of the uvula through surgery, and the procedure can be viewed in two ways [1, 2]. As a scientific treatment option to address conditions, for example, obstructive sleep apnea and hereditary angioneurotic edema [3, 4]. The second view is as part of traditional and cultural medicine due to the attitude to prevent and treat various diseases as well as considered a ritual. The traditional view can cause serious side effects, which can lead to death in children [5, 6].

Traditional uvulectomy is a surgical procedure in which the total or partial part of the uvula is removed by traditional surgeons. The uvula is a small pendant fleshy lobe that is located above the throat, hangs from the palate at a lower central border, and between the two lymphoid tissues or tonsils. Uvula helps to prevent choking, during swallowing by moving superiorly and close to the nasopharynx keep swallowed matter like food and liquid get into the nasal cavity [7–9]. There is a hypothesis suggesting that the uvula has some glandular tissue (glands) indicating that it can produce saliva which moistens the throat. It has a function in speech as well, used to articulate consonant sounds [3, 10, 11].

Traditional instruments such as a sharp blade (knife) and threads made from horsetail and other inputs are used for the cutting of uvula. First, the baby is held firmly and then the tongue is pulled. The uvula is then strapped by a thread, and cut out with a sharp instrument prepared by a traditional surgeon. The tools used to cut uvula are not clean and the procedure is careless, the individual who is subject to the practice is more likely to be exposed to a variety of health problems including death. As noted by various studies, hemorrhage following the procedure, nasal regurgitation, adjacent body part trauma (soft palate and tongue base abrasion), swallowing difficulty, malnutrition, speech problem, and local infections like abscesses, otitis media were the most prevalent complications [12–15]. And also systemic complications had been reported like septicemia, tetanus, hepatitis, HIV, and severe anemia [16–19].

Traditional uvulectomy was seen in various African countries, such as Kenya, Niger, Tanzania, Nigeria, Sudan, Eritrea, and Ethiopia [6, 20, 21]. The reasons for practicing this cultural treatment vary from country to country, and studies in different African countries at different times showed that parents and traditional surgeons were assumed uvula cause to several throat disorders and therefore, they believed it should be cut and removed [11, 22]. From a community-based cross-sectional study done in Merawi town, Amhara region of Ethiopia reported that educational status, occupation, and the previous good result were the factors associated with the practice of traditional uvulectomy [23]. A qualitative investigation in Dadar, Oromia region of Ethiopia revealed that cultural influences, presence of traditional professionals in the area, family and peer pressure, lack of knowledge, and distance from health facilities were the main reasons for uvulectomy [24].

As parents and traditional surgeons affirmed, fear of the upper respiratory tract obstruction by large uvula and that the children will be leading to death is the main reason to undergo the procedure [5, 11]. Children were the most vulnerable because consent was determined by the parents’ believe (viewpoint) and the benefit of the surgeon, not the child’s will [3, 6]. In addition, researchers had pointed out that uvula was seen as the source of throat problems in childhood end up with vomiting, feeding difficulty, and hoarseness [13, 22].

Although the age at which uvula cutting varies from country to country, it was most commonly seen in children under 5 years old and the peak age of the practice was in children 0–12 months [14, 16, 17]. In Ethiopia, 83.5% of uvulectomy was performed under 6 months of age [19].

To tackle traditional uvulectomy and the related complication, Ethiopia launched a national strategy and plan to eradicate (end) every harmful traditional practice including traditional uvulectomy by 2025 [25]. Great efforts have been done to achieve the national plans; such as health education about the negative consequences, community mobilization, and awareness creation to social help like “idir” leaders, religious leaders, and traditional healers to draw clients enhancing the capacity of service provision [25–27].

Despite the above efforts to reduce the problem over the years, findings from studies in different regions of Ethiopia show that the practice is still continuous and as many of the study participants reported that the victims were vulnerable to various health conditions. If the current situation (the practice of uvulectomy) is continuous, it will be impossible to accomplish the national plan or expectation of ending (eradicating) every traditional harmful practice from Ethiopia by 2025.

To comprehend a traditional practice of uvulectomy and achieve national expectations, localized and contextualized understanding of the practices and associated factors is crucial. Therefore, we conducted a community-based cross-sectional study to assess the practice and associated factors (identify determinants) of traditional uvulectomy among caregivers having children less than 5 years old in South Gondar Zone Amhara Region and to plan specific interventions to avoid traditional uvula cutting.

## Methods

### Study design, period, and area

A community-based cross-sectional study was conducted from March 1 to March 31, 2020, in South Gondar Zone, which is one of the Zones in the Amhara Region of Ethiopia and bordered on the South by East Gojjam, on the Southwest by West Gojjam and Bahir Dar, on the West by Lake Tana, on the North by Central Gondar, on the Northeast by Wag Hemra, on the East by North Wollo, and on the Southeast by South Wollo. The Zone is comprised of 13 woredas (Andabet, Dera, Ebenate, Estie, Farta, Fogera, Gunabegemider, Libokemkem, Lay Gayenet, Mena Meketewa, Sedie Muja, Simada, and Tachegayenet) and two administrative towns (Debre Tabor and Woreta), the capital of which is Debre Tabor, 666 km Southwest of Addis Ababa, the capital city of Ethiopia and 103 km Southeast of Bahir Dar, the capital city of Amhara Region. The total area of the Zone is 14,095.19 square kilometers and according to the 2007 census, the total population was 2,051,738, from which 1,041,061 were males and 1,010,677 females [28]. From the total population of 277, 887 were under-five children with 2:1 male to female ratio. Among the total population, 96.14 percent of the population was Orthodox religious and 3.68 percent were Muslim. The zone has one general hospital, seven district hospitals, and 93 health centers.

### Population

All caregivers who had children less than 5 years old in South Gondar Zone were the source population of this study. However, caregivers who had less than five years of old children live in selected kebeles were the study population of this study.

### Inclusion and exclusion criteria

Caregivers with children under five years of age who provided information and were available at the time of data collection were included in the study. Whereas, caregivers who were seriously ill at the time of data collection were excluded from the study.

### Sample size determination

The sample size was calculated by using a software (EPI info version 7.2.1.0) considering the assumption of a 95% confidence interval, 5% degree of precision, 80% power, unexposed to the exposed ratio of 1:1, 10% non-response rate and design effect of 2 (Table 1). Finally, the sample size was 634.

**Table 1:** Sample size calculation by using the explanatory variable.

Predictor	Assumption	Proportion in %	Initial sample size (n)	Design effect correction (2 * n)	Final sample size with a 10% non-response rate
Occupation (farmer)	Power = 80% CI = 95% 1:1 Ratio AOR = 2.859	P1 = 31.5% P2 = 8.3%	288	576	634

### Sampling procedure

**Fig 1:**
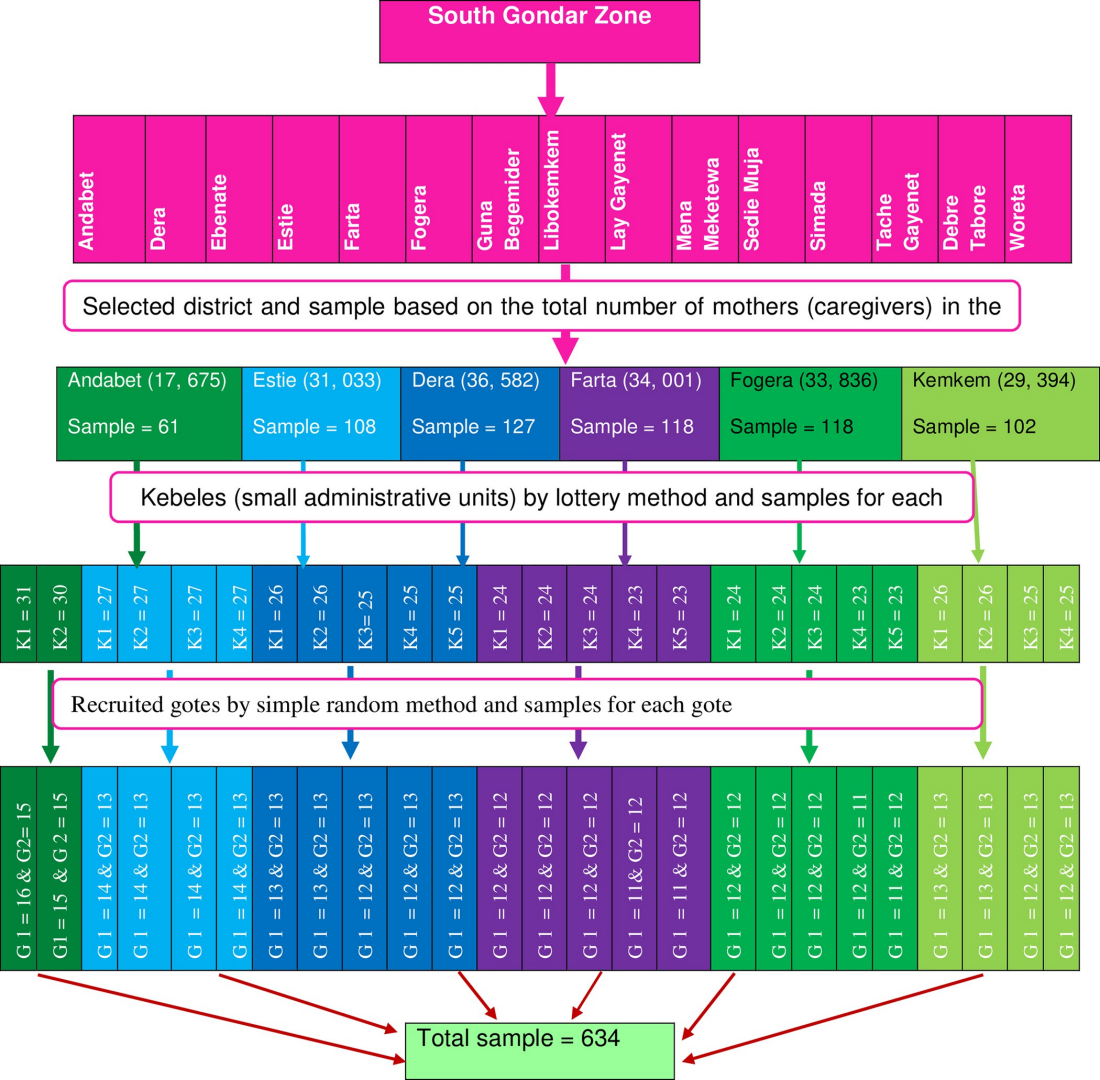
Schematic presentation of sampling procedures, caregivers having children less than 5 years old, in South Gondar Zone, Amhara Region, Ethiopia, 2020.

A multi-stage sampling technique was used to select the study participants. First, by using the lottery method six woredas were selected, and based on the total number of caregivers having children less than five years in each district the study sample proportional allocation was made. After that, of the six districts, the recruited kebeles (small administrative units) were selected by the lottery method and proportional allocation of sample for the selected kebeles was made. In addition, “Gotes” from the recruited kebeles were selected with simple random sampling. Finally, the study subject or participant caregivers were selected in each chosen “Gotes” by using a sampling frame which was obtained from the local health extension workers (Fig 1).

### Operational definitions

Knowledge: those caregivers who scored the mean or above for knowledge assessment questions were grouped as good knowledge and those caregivers who scored below the mean for knowledge assessment questions were grouped as poor knowledge.

Practice: this was measured from the report of caregivers and was coded 1 if caregivers reported that had performed traditional uvulectomy on their children; otherwise, it was coded 0.

Attitude: related issues of the respondent were assessed by five yes or no questions focusing on the perception about uvula, harmfulness of traditional uvulectomy, future intention, encouraging others to perform uvulectomy on their children, and the eradication of traditional uvulectomy. It was measured separately from the report of caregivers and coded 1 if the caregivers reported yes; otherwise, it was coded 0.

Traditional uvulectomy: surgical removal of the uvula by traditional practitioners.

### Ethical considerations

Ethical clearance was obtained from the ethical review committee of the School of Nursing on behalf of the institutional review board of the University of Gondar with *Ref*.*No*.*; -S/N/2012/06/2012*. Further permission and a supportive letter were obtained from zonal administrative, districts, and local (kebele) administrative. Ethical considerations were taken into account throughout the study. Voluntariness was asked and participants were informed as their participation is voluntary and that they can withdraw at any time of the study. In addition, the objective of the study was verified by the participants. They were informed about the confidentiality of the data being collected and oral consents were obtained. Data was collected by excluding names or any other personal identifiers from data collection tools. Moreover, the identifier for each eligible subject was replaced by a code. At the end of the interview, participants were informed about traditional uvula cutting and related issues.

### Data collection tools and procedures

Data collection tool: a structured questionnaire containing socio-demographic background, questions about traditional uvulectomy knowledge, practice, and attitude as well as associated factors which were adapted from various works of literature previously done on similar topics [13, 15–17, 19, 22, 29, 30]. The questionnaire was first prepared in English and then translated to the local language (Amharic).

Data collection procedure: the data was collected by two trained health extension workers from caregivers who have children less than 5 years old using an interviewer-administered questionnaire. Study participants having two under-five children were included with their younger children. And also, the data collection process was supervised by one clinical nurse. Instead of personal identifier code for each study participants were given. Data were checked and cleaned daily for completeness and consistency during data collection.

### Data quality control

To ensure the quality of data, intensive training was given for 1 day before the data collection procedure starts to data collectors and supervisors about data collection processes and techniques. Local language-translated tools were used. A pre-test was conducted on 32 eligible caregivers (5% of sample size) at Andabet woreda Jaragedo and Semete kebele and that was not included in the study and tool modification was made by adding a variable health personnel hospitality problem. Moreover, the data collection process was supervised strictly.

### Data processing and analysis

Data were entered and edited using Epi-Data by the principal investigator and then exported into SPSS version 23 for analysis. Descriptive measures of statistics like the frequency with percentage, mean with standard deviation, and median with interquartile range was used to describe the socio-demographic characteristics and the practice of traditional uvulectomy. Logistic regression (both bivariable and multivariable) was performed to identify the factors associated with traditional uvula cutting. Variables with a p-value < 0.2 in the binary regression analysis were entered for multivariable analysis and variables that had p-value < 0.05 in final model were reported as associated factors with adjusted odds ratio (AOR) and 95% CI. To check the model goodness of fit Hosmer and Lemeshow test was used.

## Results

### Socio-demographic characteristics

In this study, a total of 634 caregivers who had children less than five years were included with a response rate of 100%. The mean age of participant caregivers was 28.78 years (SD = ± 4.352). The median (IQR) age of children was 16 (9 to 25) months. The majority of the respondents, 605 (95.4%) were Orthodox religious followers. Nearly all the caregivers, 604 (95.3%) were married. Regarding occupational status about 354 (55.8%) of respondents were farmers. About educational status, 335 (52.8%) could not read and write. The majority of the respondent’s family monthly income was in between 2131–3560 ETB (Table 2).

**Table 2:** Socio-demographic characteristics of caregivers having less than five years old children in South Gondar Zone, Amhara region, Ethiopia, 2020 (N = 634).

Variables		Frequency (N)	Percentage (%)
**Caregivers age in years**	20–24	99	15.6
	25–29	330	52.1
	30–34	122	19.2
	35 and above	83	13.1
**Child age in months**	1–6	104	16.4
	7–12	145	22.9
	13–18	140	22.1
	19–24	84	13.2
	25–30	53	8.4
	31–36	33	5.2
	37–42	24	3.8
	43–48	24	3.8
	49 and above	27	4.3
**Religion**	Orthodox	605	95.4
	Muslim	29	4.6
**Marital status**	Married	604	95.3
	Divorced	30	4.7
**Educational status**	Cannot read and write	335	52.8
	Can read and write	13	2.1
	Elementary school	113	17.8
	High school and preparatory	77	12.1
	College diploma and above	96	15.1
**Occupational status**	Farmer	354	55.8
	Housewife	101	15.9
	Civil servant	88	13.9
	Daily laborer	11	1.7
	Trader (merchant)	80	12.6
**Family monthly income in ETB**	700–2130 ETB	139	21.9
	2131–3560 ETB	203	32.0
	3561–4990 ETB	137	21.6
	4991–6420 ETB	96	15.1
	6421–7850 ETB	36	5.7
	7851 ETB and above	23	3.6

### Knowledge about traditional uvula cutting and related issues

Out of the 634 respondents, 431 (68%) had information about traditional uvulectomy, but the overall participants who had good knowledge were 392 (61.8%). The major sources of information were family members 410 (64.7%) and neighbors 400 (63.1%), (Table 3).

**Table 3:** Knowledge of caregivers having less than five years old children about traditional uvulectomy in South Gondar Zone, Amhara region, Ethiopia, 2020 (N = 634).

Variable			Frequency (N)	Percentage (%)
**Information about traditional uvulectomy**	Yes		431	68
	No		203	32
**Source of information**	Health personnel		141	22.2
	Family member		410	64.7
	Neighbors		400	63.1
	Book and at school		80	12.6
	Meeting		38	6
	Social media		33	5.2
	Others*		8	1.3
**Information by type**	Uvulectomy is bad practice	Yes	431	68
		No	203	32
	Uvula function	Yes	83	13.1
		No	551	86.9
	About the health risk of uvula cutting	Yes	431	68
		No	203	32
	About available modern treatment options	Yes	392	61.8
		No	242	38.2
	About available home remedies	Yes	84	13.25
		No	550	86.75

*Others; friends and traditional surgeons

### The practice of traditional uvulectomy in the South Gondar zone

**Fig 2:**
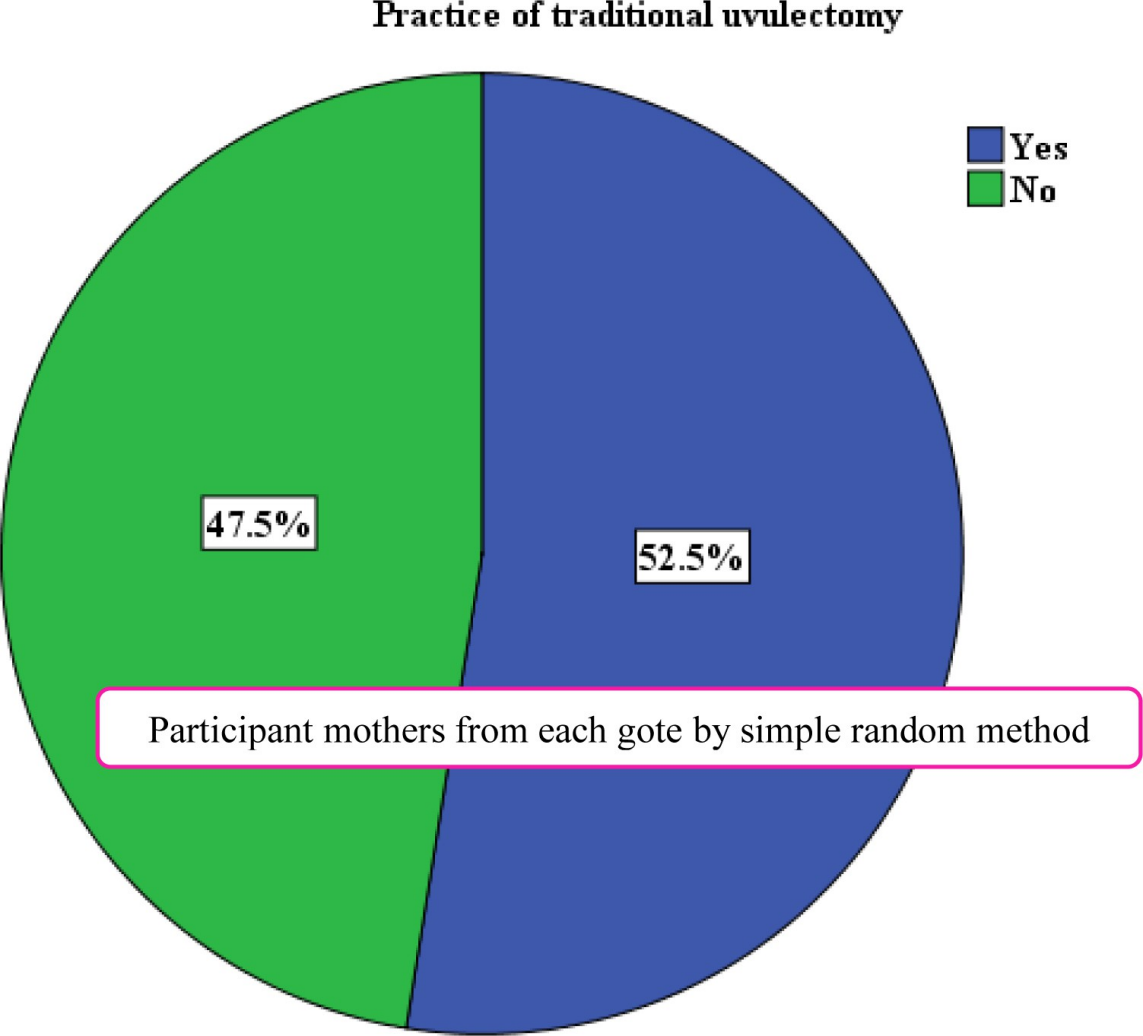
Practice of traditional uvulectomy among caregivers having children less than five years old in South Gondar Zone, Amhara region, Ethiopia, May 2020.

The result of the study showed that about 333 (52.5%) (95% CI = 48.6–56.3%) caregivers practiced traditional uvula cutting to their children for having less than five years old (Fig 2). The median (IQR) age of uvula cutting was 5 (1/2 to 9) months. Among those children subject to traditional uvula cutting 39 (11.71%) faced complications following the procedure and the complication were adjusted organ damage such as throat and teeth (Table 4).

**Table 4:** Uvula cutting practice among caregivers having children less than five years old in South Gondar, Amhara Region, Ethiopia, 2020 (N = 634).

Variables			Frequency (N)	Percent (%)
**Traditional uvulectomy**	Yes		333	52.5
	No		301	47.5
**Age of child in days while the uvula cut**	2–182		189	56.8
	183–452		90	27.0
	453–900		54	16.2
**Who decided to practice uvulectomy?**	The mother of the child		299	89.8
	The father of the child		285	85.6
	Ancestor (grandfather and/or mother)		120	36
	Others*		59	17.72
**Complication following uvulectomy**	Yes		39	11.71
		Adjust organ damage such as throat and teeth	39	11.71
	No		294	88.29

*others; neighborhood and friends

### Attitude related issues about uvulectomy

The majority, 410 (64.7%) of the study participant had perceived as uvula causes illness and 365 (57.6%) of the study subject perceived as traditional uvula cutting is not harmful. And also 408 (64.4%) of the study participant believed that traditional uvula cutting should not be eradicated and the main reason was it is effective 375 (59.1%), (Table 5).

**Table 5:** Attitude related issues about uvulectomy among caregivers having children less than five years old in South Gondar, Amhara Region, Ethiopia, 2020 (N = 634).

Variables			Frequency (N)	Percentage (%)
**Perceived as uvula causes illness**	Yes		410	64.7
	No		224	35.3
**To what disease does uvula causes**	To sore throat		410	64.7
	Fever		311	49.1
	Swallowing difficulty		410	64.7
	Behavioral change		154	24.3
	Vomiting		71	11.2
	Others *		42	6.6
**Perceived traditional uvula cutting as harmful**	Yes		269	42.4
	No		365	57.6
**Will perform traditional uvula cutting**	Yes		311	49.1
	No		323	50.9
**Encourage others to perform traditional uvulectomy**	Yes		310	48.9
	No		324	51.1
**Traditional uvula cutting should be eradicated**	Yes		226	35.6
	No		408	64.4
**Why not eradicated**	Against culture	Yes	154	24.3
		No	480	75.7
	It is effective	Yes	375	59.1
		No	259	40.9

*others; snoring, to failure to thrive, and diarrhea

### Reasons to practice traditional uvulectomy

**Fig 3:**
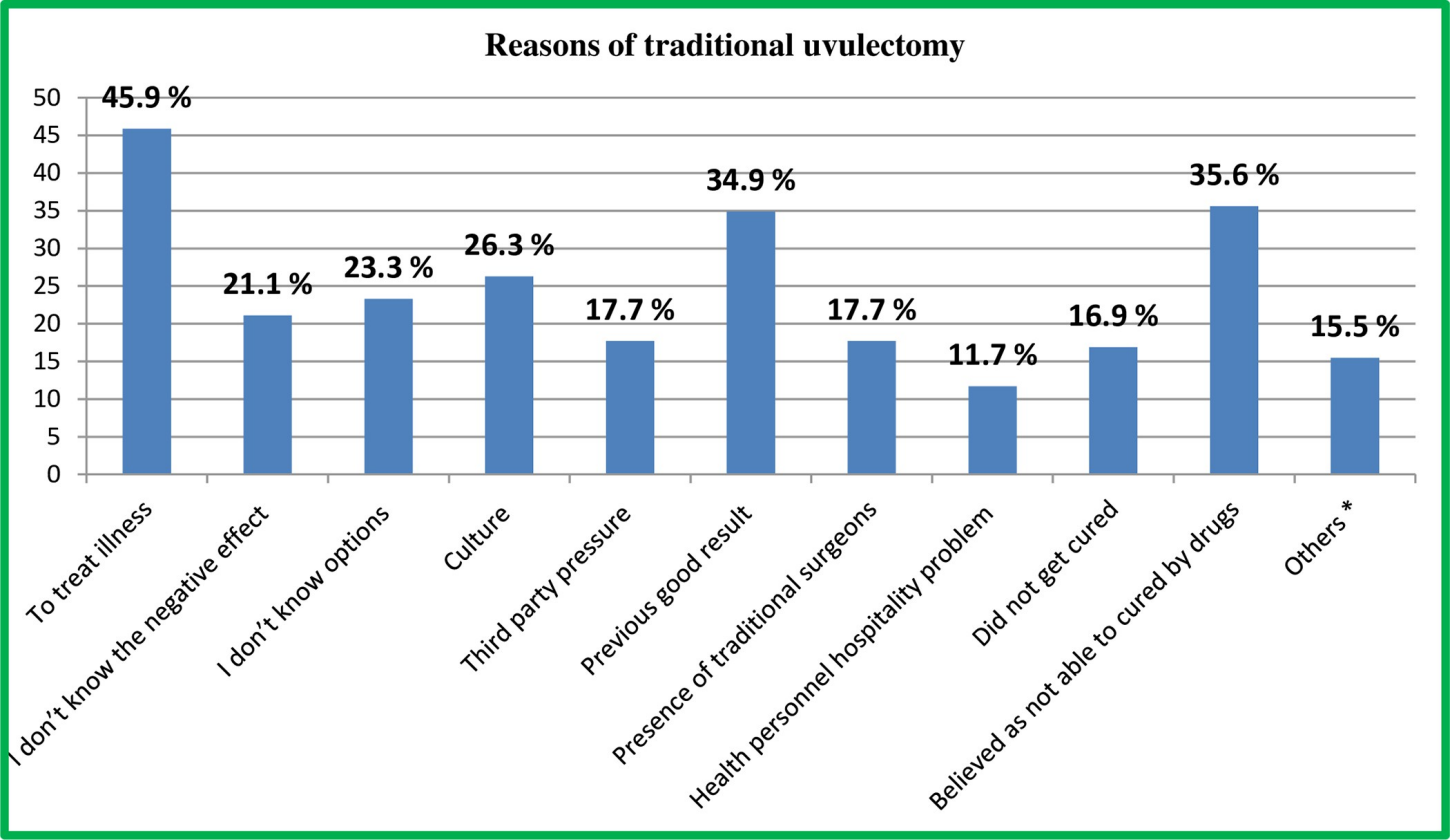
Reasons for traditional uvulectomy among caregivers having children less than five years old in South Gondar Zone, Amhara region, Ethiopia, May 2020. **N.B**: Others*; early prevention, less cost, no nearby health facility, and family history of uvulitis.

The main reasons to subject their child for traditional uvula cutting were to treat illness (sore throat and difficulty of swallowing as well as recurrent infection), perceived as not able to cure by drugs and a previous good result as mentioned by 291 (45.9%), 226 (35.6%) and 221 (34.9%) of the study participant caregivers, respectively (Fig 3).

### Factors associated with traditional uvula cutting

In bivariate analysis; child age, caregiver occupational status, family monthly income, poor knowledge (no information about traditional uvulectomy and related issues), perceived as uvula causes illness, perceived as an ultimate treatment option, future intention (will perform traditional uvula cutting), encourage others to practice uvula cutting, perceived as traditional uvula cutting is not harmful, perceived as traditional uvulectomy should not be eradicated, saw the previous good result, didn’t get cured by pharmacologic treatment and health personnel hospitality problem were statistically associated with traditional uvulectomy with p-value <0.05 at 95% C.I. (Table 6).

**Table 6:** Bivariable and multivariable logistic regression analysis of factors associated with traditional uvulectomy among caregivers having children less than 5 years old in South Gondar Zone, Amhara Region, Ethiopia, 2020 (N = 634).

Variables		Traditional uvulectomy			
		Yes	No	COR 95% CI	AOR 95% CI
**Child age in months**	1–6	50	54	.637 (.270, 1.502)	
	7–12	85	60	.974 (.422, 2.247)	
	13–18	74	66	.771 (.334, 1.779)	
	19–24	47	37	.873 (.362, 2.106)	
	25–30	30	23	.897 (.350, 2.297)	
	31–36	13	20	.447 (.158, 1.261)*	
	37–42	12	12	.687 (.227, 2.084)	
	43–48	6	18	.229 (.069, .762)**	
	49 and above	16	11		
**Educational status**	Cannot read and write	193	142	1.417 (.899, 2.234)*	
	Can read and write	7	6	1.216 (.381, 3.886)	
	Primary school	56	57	1.024 (.594, 1.765)	
	High school and preparatory	30	47	.665 (.362, 1.223)*	
	College diploma & above	47	49		
**Occupational status**	Farmer	197	157	1.787 (1.093, 2.923)**	
	Housewife	57	44	1.845 (1.019, 3.342)**	
	Civil servant	42	46	1.300 (.706, 2.395)	
	Daily laborer	4	7	.814 (.220, 3.006)	
	Trader (merchant)	33	47		
**Family monthly income**	700–2130 ETB	77	62	2.329 (.927, 5.849)*	
	2131–3560 ETB	107	96	2.090 (.849, 5.146)*	
	3561–4990 ETB	79	58	2.554 (1.015, 6.425)**	
	4991–6420 ETB	48	48	1.875 (.727, 4.832)*	
	6421–7850 ETB	14	22	1.193 (.402, 3.544)	
	7851 ETB and above	8	15		
**Information about uvulectomy**	Yes	199	232		
	No	134	69	2.264 (1.601, 3.202)****	2.975 (1.677, 5.277)****
**Uvula causes illness**	Yes	261	149	3.698 (2.619, 5.222)****	4.888 (2.954, 8.086)****
	No	72	152		
**Harmful**	Yes	87	110		
	No	246	191	1.608 (1.171, 2.208)***	
**Will perform**	Yes	204	107	2.867 (2.076, 3.960)****	4.188 (2.584, 6.788)****
	No	129	194		
**Encourage others**	Yes	177	133	1.433 (1.048, 1.960)**	
	No	156	168		
**Should be eradicated**	Yes	89	137		
	No	244	164	2.290 (1.643, 3.193)****	1.893 (1.172, 3.057)***
**To treat illness**	Yes	178	133	1.911 (1.391, 2.625)****	
	No	155	188		
**Saw the previous good result**	Yes	185	36	9.201 (6.108, 13.862)****	9.396 (5.512, 16.016)****
	No	148	265		
**Hospitality problem**	Yes	59	15	4.106 (2.275, 7.410)****	5.922 (2.392, 14.664)****
	No	274	286		
**Didn’t get cured by drug treatment**	Yes	78	29	2.869 (1.813, 4.541)****	3.918 (2.073, 7.405)****
	No	255	272		

N.B

*P< 0.2

**P < 0.05

***p < 0.01

****p < 0.001

After bivariable analysis, those variables with p-value <0.2 were entered for further multivariable analysis. After adjusting for potential confounders in multivariable logistic regression analysis; no information about traditional uvula cutting and related issues, perceived as uvula causes illness, future intention (will perform traditional uvula cutting), perceived as traditional uvulectomy should not be eradicated, saw the previous good result, health personnel hospitality problem and didn’t get cured by pharmacologic treatment were significantly related with traditional uvulectomy. But child age, caregiver’s educational status, caregivers’ occupational status, family monthly income, and perceived as traditional uvulectomy is not harmful weren’t significantly associated with traditional uvulectomy in multivariable analysis.

Caregivers who had no information about traditional uvulectomy and related issues were 2.975 times more likely to practice traditional uvulectomy in their children than those who have information about traditional uvula cutting and related issues [AOR = 2.975 (p < 0.001, 1.677–5.277)].

Caregivers who perceived as uvula causes illness were 4.888 times more likely to subject their child for traditional uvulectomy [AOR = 4.888 (p < 0.001, 2.954–8.086)] than those who were perceived as not causes illness.

Similarly, future intention (will perform) was significantly associated with traditional uvulectomy. Caregivers who claimed to perform traditional uvula cutting in the future were 4.188 times more likely to practice uvula cutting traditionally [AOR = 4.188 (p < 0.001, 2.584–6.788)] than those who not intended to practice in the future.

Caregivers who perceived as traditional uvulectomy should not be eradicated were 1.893 more likely to practice traditionally uvulectomy on their children than those who perceived as it should be eradicated [AOR = 1.893 (p = 0.009, 1.172–3.057)].

Those caregivers who saw the previous good result practiced traditional uvulectomy on their children 9.396 times than those who didn’t see previous good results [AOR = 9.396 (p < 0.001, 5.512–16.016)].

There was a strong association also between complaints of health personnel hospitality problems and practice of traditional uvulectomy on children. As a result, caregivers who complained of health personnel hospitality problems were 5.922 more likely to practice uvulectomy on their child than those who didn’t complain about health personnel hospitality problems [AOR = 5.922 (p < 0.001, 2.392–14.664)].

Regarding therapeutic response, caregivers who claimed didn’t get cured by pharmacologic treatment practices traditional uvulectomy on their children 3.918 times than caregivers not claimed didn’t get cured by pharmacologic treatment [AOR = 3.918 (p < 0.001, 2.073–7.405)].

## Discussion

In this study, we aimed to assess the practice of traditional uvulectomy and its associated factors among caregivers having children less than 5 years old in the South Gondar zone, Amhara Region, Ethiopia. Consequently, the practice of traditional uvulectomy was found to be 52.2% (95% CI: 48.6–56.3%) and lack of information, perceived as uvula causes illness, future intention, perceived as uvulectomy should not be eradicated, saw the previous good result, health personnel hospitality problem and didn’t get cured by pharmacologic treatment were the factors significantly associated with traditional uvulectomy.

The prevalence of uvulectomy in this study was lower than in a study conducted in Plateau State (Jos) and Jigawa State Nigeria which was practiced by 86.1% and 90% of the respondents, respectively [13, 16]. The difference might be cultural disparities between the studies in Nigeria uvulectomy which was considered as religious dictates and making ritual as well as naming ceremony.

In addition, this was lower than a study conducted in Axum (which ranges from 72.8% to 86.9%) and in Fentale Woreda 84.7% [14, 29, 31]. The variation might be because of the time gap between the studies and it may improve awareness about scientific treatment options of uvulitis as well as increase the accessibility of health care services. But it was higher when compared to a research done in Debre Birhan Town (23.7%) and Merawi town (20%) [23]. This difference might be because of the variation of the study settings. In this study, participants were residing in the rural area as a result of less access to the health facility and sources of information like social media; on the contrary easily accessible to traditional surgeons.

In this study, the main reasons to perform traditional uvulectomy mentioned by caregivers were to treat illness (sore throat and difficulty of swallowing as well as recurrent infection) (45.9%), perceived as not able to cure by drugs (35.6%), and a previous good result (34.9%). On the contrary to this study, a study carried out in Nigeria, suggested that a majority of participants(65.5%) did not know why they have undergone uvulectomy [13]. This variation might be due to the difference of study population and methodology, in which the respondents of the previous study were the individuals with amputated uvula in the age range of 2 to 53 years old and conducted by direct examination of the participant for presence or absence of their uvula (observational study). As a result, they may not remember what was the indication for the procedure being performed on them or did not know the indication since practiced on their childhood age by the consent of parents or caregivers.

The reasons affirmed by this study were different in Axum where the main reason was the early prevention of swelling (68.5%) [14, 29]. This might be because of awareness of caregivers towards the treatment option of uvulitis has been improved as a result they may first attempt scientific treatment.

Adjusting for other factors, lack of information was significantly associated with traditional uvulectomy practice. Caregivers who had no information about traditional uvula cutting and related issues were 2.975 times more likely to practice traditional uvulectomy in their children than those who have information about traditional uvulectomy and related issues. This is true that as awareness about available treatment options and the negative effect of traditional uvulectomy improved, the tendency towards seeking scientific treatment be increased.

Caregivers’ attitudes-related concerns were other pushing factors to practice traditional uvulectomy. In this study, among the total respondents, 410 (64.7%) caregivers perceived as uvula causes illness was found to be significantly associated with traditional uvula cutting. Those caregivers who were perceived as uvula causes illness were 4.888 times to subject their child for traditional uvulectomy than those who were perceived as not causing illness. This is nearly consistent with a previous study done in central rural Tanzania where 90.3% of the respondents believed that long uvula (uncut uvula) causes illness (prolonged cough and fever) [32]. This is true that attitude undermined the practice as a result if a caregiver perceived as uvula causes illness, they are more adduce to practice uvulectomy.

Caregivers perceived as traditional uvulectomy should not be eradicated (since effective) was another factor significantly related to uvulectomy. In this study, 408 (64.4%) of participants perceived as traditional uvulectomy should not be eradicated (because it is effective = 308 (75.49%). Adjusting for other factors, those caregivers who perceived as traditional uvula cutting should not be eradicated were 1.893 more likely to practice traditional uvulectomy on their children than those who perceived as it should be eradicated. This was in agreement with a study done at the Congolese refugee camp in Tanzania where uvulectomy is beneficial and effective than modern medicine [33]. Negative attitudes undermined the healthcare-seeking behavior and pull to practice what they perceived as a result if the caregivers perceived that traditional uvulectomy is effective, they are more likely to practice it.

Future intention (will perform) was also significantly associated with traditional uvulectomy. Caregivers who claimed to perform traditional uvula cutting in the future were 4.188 times more likely to practice traditional uvulectomy than those who did not intend to practice in the future. This is true that a volition that intends to carry out is a driving force to attempt it.

Those caregivers who saw previous good results practiced uvula cutting on their children 9.396 times than those who didn’t see previous good results. This study is similar to a study conducted in the Merawi Amhara region, Ethiopia, where previous good outcomes [23]. This could be based on if caregivers saw the good result from the previous uvulectomy procedure; they accept it as an effective and an alternative option and are more likely to practice it.

Another factor pointed out by this study that affects uvulectomy was health personnel hospitality problems. Among caregivers who subject their child to uvulectomy, 74 (11.7%) participants complained health personnel hospitality problem was the pushing factor. Moreover, caregivers who complained of health personnel hospitality problems were 5.922 more likely to practice uvulectomy on their child than those who didn’t complain of health personnel hospitality problems. This was in line and nearly similar to reasons mentioned in a study carried out in Axum in which a lack of better medical care as a pushing factor was stated by 12.8% of respondents [29]. This association could be due to hospitality problem that may lead to dissatisfaction and reduce the likelihood of healthcare-seeking behavior and pushes them to traditional uvulectomy.

Didn’t get cured by pharmacologic treatment was another factor pointed out by this study. Among the respondents who subject their child to uvula cutting 107 (16.9%) claimed that didn’t get cured by pharmacologic treatment was the reason to practice traditional uvulectomy. Adjusting other factors, caregivers who claimed didn’t get cured by pharmacologic treatment practices uvula cutting traditionally on their children 3.918 times than caregivers not claimed didn’t get cured by pharmacologic treatment. This was in line and lower than the study carried out in Debre Birhan Town in which 50.5% claimed no cure with modern medicine [15].

As there were few studies conducted on traditional uvulectomy, this study might have an input for further research and will be used as a baseline data. It might also imply child health care by identifying the gaps it helps to reduce child morbidity and to improve quality of care.

## Limitations

Even though the study conducted is community-based than institution-based to have a more representative prevalence of traditional uvulectomy, data obtained from the study participants through self-report were not cross-checked with their actual practices on the ground and clinical observation of the throat. In addition, the study didn’t include a qualitative method as a result factor associated with traditional uvulectomy other than those factors described in the literature may not be addressed.

## Conclusion

The prevalence of uvula cutting was found to be high (since traditional uvulectomy is forbidden and there is a national plan to eradicate it by 2025). The factors associated with traditional uvulectomy were lack of information about traditional uvula cutting and related issues, perceived as uvula causes illness, future intention or will perform traditional uvula cutting, perceived as traditional uvulectomy should not be eradicated (it is effective), saw the previous good result, health personnel hospitality problem and did not get cured by pharmacologic treatment. Better to create further awareness to the community at large and set controlling mechanisms to the health care delivery system.

## Acronyms and/or abbreviations

AIDS: Acquired Immune Deficiency Syndrome
AOR: Adjusted Odds Ratio
CI: Confidence Interval
COR: Crude Odds Ratio
ETB: Ethiopian Birr
HIV: Human Immunodeficiency Virus
IQR: Inter Quartile Range
OR: Odds Ratio
SD: Standard Deviation
TV: Television

## References

[1] Taber’s cyclopedic medical dictionary. 22 edition ed: F. A. Davis Company; 2013. p. 2427.

[2] Mosby’s Dictionary of Medicine, Nursing & Health Professions, 1oth Edition Ed: Elsevier Inc.; 2017. P. 1842.

[3] Kawia HM, Kahabuka FK, Mbawalla HS. Parental deceptive information: A case of traditional uvulectomy. Tanzania Dental Journal. 2014;18(2):76–80.

[4] Sa Khalam, Nm Kurien. Uvulectomy; Symptomatic relief for chronic irritating cough and obstructive apnoea syndrome in long uvula. Journal of Case Reports. 2014;2(1):9–11.

[5] Agbor AM, Naidoo S. A review of the role of African traditional medicine in the management of oral diseases. African Journal of Traditional, Complementary and Alternative Medicines. 2016;13(2):133–42.

[6] Protecting children from harmful practices in plural legal systems with a special emphasis on Africa. New York: Save the children’s, 2012.

[7] Barbara Janson Cohen KLH. Memmler’s Structure and Function of the Human Body, 11th edition. 2016.

[8] Sons JW. Fundamentals of Anatomy and Physiology For Nursing and Healthcare Students, Second edition. 2017.

[9] Hoehn ENMK. Human Anatomy & Physiology, tenth edition. 2016.

[10] Tf Encyclopedia. Palatine uvula. Palatine uvula 16 November 2019.

[11] Olajide GT, Olajuyin OA, Adegbiji Wa. Traditional Uvulectomy: Origin, Perception, Burden, And Strategies of Prevention. International Journal of Medical Reviews and Case Reports. 2018:1–4.

[12] Ajiya A. Pattern of otitis media in young children and adolescents with traditional uvulectomy in Kano, Nigeria. Annals of African Medical Research. 2019;2(2).

[13] Adoga AA, Nimkur TL. The traditionally amputated uvula amongst Nigerians: still an ongoing practice. ISRN otolaryngology. 2011;2011. doi: 10.5402/2011/704924 23724258 PMC3658569

[14] Gebrekirstos K, Fantahun A, Buruh G. Magnitude and reasons for harmful traditional practices among children less than 5 years of age in Axum Town, North Ethiopia, 2013. International Journal of pediatrics. 2014;2014. doi: 10.1155/2014/169795 25045359 PMC4089850

[15] Kabtamu Kebede KM, Msanew Chekole, Amakelew Zewdie, Hirut Redahegn, Alebachew Demelash, and Sisay wasinad. Prevalence and Associated Factors to Uvula Cutting on Under Five Children in Amhara Region, Debre Birhan Town. International Journal of Pediatrics and Child Health. 2016/17;5.

[16] Ajibade B, Okunlade J, Kolade O. Harmful cultural practices: parents perceived effects of traditional uvulectomy on the under-five-children in Jigawa State, Nigeria. Journal of Dental and Medical Sciences. 2013;9(5):8–13.

[17] Abdullahi M, Amutta S. Traditional Uvulectomy Among The Neonates: Experience In A Nigerian Tertiary Health Institution. Borno Medical Journal. 2016;13(1):16–20.

[18] Kambale RM, Balibuno Y, Francisca NI, Kasengi JB, Mayele GF, Masumbuko BM. Traditional uvulectomy, a common practice in South Kivu in the Democratic Republic of Congo. Medecine et sante tropicales. 2018;28(2):176–81. 29997076 10.1684/mst.2018.0779

[19] Mitke YB. Bloody traditional procedures performed during infancy in the oropharyngeal area among HIV+ children: implication from the perspective of mother-to-child transmission of HIV. AIDS and Behavior. 2010;14(6):1428–36. doi: 10.1007/s10461-010-9681-4 20217469

[20] Thomson M. Rights of Passage. Harmful cultural practices and children’s rights. 2003.

[21] Assembly G. Report of the Committee on the Rights of the Child. Official RecordsSixty-seventh session supplement No 41 (A/67/41). 2012.

[22] Isa A, Omotara B, Sandabe M, Garandawa H. Parental reasons and perception of traditional uvulectomy in children. Sahel Medical Journal. 2011;14(4):210.

[23] Wassie SM, Aragie LL, Taye BW, Mekonnen LB. Knowledge, Attitude, and Utilization of Traditional Medicine among the Communities of Merawi Town, Northwest Ethiopia: A Cross-Sectional Study. Evidence-Based Complementary and Alternative Medicine. 2015;2015:7. doi: 10.1155/2015/138073 26508974 PMC4609866

[24] Elias A, Tesfaye G, Bizatu M. Aspects of common traditional medical practices applied for under-five children in Ethiopia, Oromia region, eastern-Harargie district, Dadar Woreda. Commun Med Health Educ. 2013;3(6):1–8.

[25] Federal Democratic Republic of Ethiopia Ministry of Women CaYAM. National Strategy and Action Plan on Harmful Traditional Practices (HTPs) against Women and Children in Ethiopia. Addis Abeba 2013.

[26] Pankhurst A, Woldehanna T, Araya M, Tafere Y, Rossiter J, Tiumelissan A, et al. Young Lives Ethiopia: Lessons from longitudinal research with the children of the millennium: Young Lives; 2018.

[27] Ethiopia TFDRo. Fifth National Report on Progress made in the implementation of the Beijing Declaration and Platform for Action (Beijing +25). 2019.

[28] Population and Housing Census of Ethiopia. Central statistical agency, 2007; http://www.csa.gov.et/census-report/complete-report/census-2007?Start=5

[29] Gebrekirstos K, Abebe M, Fantahun A. A cross-sectional study on factors associated with harmful traditional practices among children less than 5 years in Axum town, north Ethiopia, 2013. Reproductive health. 2014;11(1):46. 24952584 10.1186/1742-4755-11-46 PMC4082281

[30] Farouk ZL, Slusher TM, Danzomo AA, Slusher IL. Factors Influencing Neonatal Practice in a Rural Community in Kano (Northern), Nigeria. Journal of tropical pediatrics. 2019. 10.1093/tropej/fmz012 30907422

[31] Kufa A. Harmful traditional practices (HTP). An analysis of its prevalence of and associated factors among children in Ethiopia. 2019.

[32] Owibingire SS, Kamya ER, Sohal KS. Beliefs about Traditional Uvulectomy and Teething: Awareness and Perception among Adults in Tanzanian Rural Setting. Annals of International Medical and Dental Research. 2018;4(2):25.

[33] Kunii O, Tanaka Y, Lewis A, Wakai S. Uvulectomy And Other Traditional Healing Practices: Traditional Healers ‘Perceptions And Practices In A Congolese Refugee Camp In Tanzania. Tropical medicine and health. 2006;34(4):159–66.

